# Species and genetic diversity of nontuberculous mycobacteria in suspected tuberculosis cases in East Azerbaijan, Iran: a cross-sectional analysis

**DOI:** 10.3389/fcimb.2024.1477015

**Published:** 2024-10-24

**Authors:** Mehdi Roshdi Maleki

**Affiliations:** Department of Microbiology, Malekan Branch, Islamic Azad University, Malekan, Iran

**Keywords:** genetic diversity, nontuberculous mycobacteria, species, suspected tuberculosis, NTM infection, diagnostic

## Abstract

**Introduction:**

The incidence of nontuberculous mycobacterial (NTM) infections has increased worldwide, attracting attention in routine diagnostic settings, particularly among patients with suspected tuberculosis. This study aimed to acquire knowledge of NTM infections in patients with suspected tuberculosis and to evaluate the genetic diversity of the strains.

**Methods:**

In this study, 230 clinical specimens were collected from suspected tuberculosis patients. Following decontamination with N-Acetyl-L-cysteine–sodium hydroxide (NALC-NaOH), the sediments of specimens were inoculated onto Löwenstein–Jensen medium and then incubated at 37°C for 8 weeks. The samples that yielded positive cultures were evaluated through the sequencing of conserved fragments of *IS6110* and *hsp65*. For those samples that were not identified as part of the *M. tuberculosis* complex (MTC) by *IS6110* PCR, further analysis was conducted via PCR to detect fragments of the *hsp65* gene.

**Results:**

Twenty-one NTM species were isolated from 230 clinical specimens (14 NTM from pulmonary specimens and 7 from extrapulmonary specimens). Among these, 12 (57.14%) were rapid-growing mycobacteria (RGM), and 9 (42.85%) were slow-growing mycobacteria (SGM). No *M. avium* complex (MAC) was identified in any of the specimens. Notably, the *M. kansasii*, *M. gordonae*, and *M. abscessus* strains presented significant genetic diversity.

**Conclusions:**

The prevalence of infections attributed to nontuberculous species surpasses that attributed to tuberculosis. These findings underscore the importance of exploring NTM species in individuals suspected of having TB.

## 1 Introduction

The genus Mycobacterium contains two major human pathogens: Mycobacterium (M.) tuberculosis (MTB), the main cause of tuberculosis (TB), and *M. leprae*, the agent responsible for leprosy. In addition to these two obligate human pathogens, the genus also includes nontuberculous mycobacteria (NTM) or Mycobacteria other than *M. tuberculosis* (MOTT) (Zulu et al., 2021).

Nontuberculous mycobacteria (NTM) are a diverse group of opportunistic pathogenic bacteria commonly found in the environment. To date, more than 170 species of NTM have been identified, with approximately one-third of these species associated with human diseases (Salzer et al., 2020; Falkinham, 2021). A study investigating the presence of NTM in the water plies of hemodialysis centers in Tabriz, Iran, analyzed 65 water samples from four hospitals and revealed a 21.5% contamination rate with NTM. Researchers have isolated 18 NTM colonies representing seven different species, with *M. fortuitum* and *M. gordonae* being the most prevalent. These findings indicate a predominance of rapidly growing mycobacteria (RGM) over slowly growing mycobacteria, including several potentially pathogenic species that pose infection risks to immunocompromised patients. This study emphasized that hemodialysis water supplies can be a source of NTM contamination, highlighting the need for continuous monitoring and improved water disinfection procedures to safeguard vulnerable populations. Additionally, sequencing the *hsp65* gene was more effective than *16S rRNA* gene sequencing for the precise identification of NTM species. Overall, these findings highlight the importance of recognizing NTM as part of the microbiological flora in hemodialysis water systems and implementing preventive measures to protect patients (Roshdi Maleki et al., 2019). In recent years, the incidence of NTM-related diseases has significantly increased (Yu et al., 2017). The lungs are the most common site of NTM infection, but other parts of the body, such as soft tissues, blood, lymph nodes, and the skin, can also be affected (Desai and Hurtado, 2021). NTM infections are usually believed to be a result of environmental exposures, including water, soil, and dust sources. A recent study examined the prevalence of NTM in the hospital water distribution systems of Tabriz, Iran, via sequence analysis of the *hsp65* and *16S rRNA* genes for species identification. The results revealed that 63.3% of the 120 water samples collected were contaminated with NTM, a notably high percentage compared with that reported in similar studies in other regions. The most common species identified was *M. gordonae*, which was found in all the water sources sampled, along with other pathogenic species, such as *M. kansasii* and *M. chelonae*. Rapid-growing mycobacteria (RGM) such as *M. mucogenicum* and *M. fortuitum*, are particularly prevalent, especially in water- cooler reservoirs. This study underscores the significant risk posed by NTM-contaminated water to immunocompromised patients, such as those undergoing bone marrow or organ transplants, and highlights the need for rigorous water quality monitoring and effective decontamination procedures in hospital settings. Although *16S rRNA* gene sequencing is common for identifying NTM, supit may be insufficient for closely related species, making the combined use of *hsp65* and *16S rRNA* gene sequences a more accurate method. The high prevalence of NTM suggests that enhanced preventive measures, such as water filtration and antimicrobial prophylaxis for vulnerable patients, are crucial for reducing the risk of nosocomial infections (Maleki et al., 2017).

Nontuberculous mycobacterial pulmonary disease (NTM-PD) was initially identified as a concomitant infection among a small subset of patients with tuberculosis (TB) sanatoria. Currently, NTM-PD poses a significant challenge because its incidence is increasing, but the reasons for this trend remain unclear. The NTM species responsible for pulmonary disease exhibit regional variability, with the *M. avium-intracellulare* complex (MAC) emerging as the primary pathogen in many areas (**Table 1**) (Cook, 2010; Cowman et al., 2019).

**Table 1:** Nontuberculous mycobacteria that cause pulmonary disease: observations on regional prevalence.

NTM Classification	Comments on regional disease prevalence
Slowly growing NTM	
*M. avium–intracellulare* complex (MAC)	Most common NTM in most regions
*M. kansasii*	Central USA, England, Wales, France
*M. malmoense*	The UK, northern Europe, rare in the USA
*M. simiae*	Arid regions of southwestern USA, Cuba, Israel
*M. xenopi*	Northern USA, Canada, UK, Paris, some regions of Europe
Rapidly growing NTM	
*M. abscessus* complex (MABC),	Regional epidemiology is less well understood; second to fourth most common NTM in some regions. Increasing in patients with cystic fibrosis (*M. abscessus*). Increasing RGM speciation, with molecular characterization.

NTMs can be classified into four types according to their growth rate and pigment formation (**Table 2**). Types I, II, and III strains are classified as slow-growing mycobacteria (SGM) because they take more than seven days to form visible colonies on a culture plate. They differ in their ability to produce pigments. The most significant species included *M. kansasii*, *M. malmoense*, *M. simiae*, *M. marinum*, and *M. xenopi*. Type IV strains are considered rapid-growing mycobacteria (RGM) because they take less than seven days to form visible colonies in culture. The most clinically important species in this category are the *M. abscessus* complex (MABC), *M. chelonae*, and *M. fortuitum* (Porvaznik et al., 2017).

**Table 2:** Classification of NTMs according to Runyon and their reported pathogenesis in humans.

Runyon Classification	NTM Species (e.g.)	Pathogenesis
Photochromogens Runyon type I	*M. kansasii* *M. simiae*	Pulmonary infections Skin infections Disseminated infections
Scotochromogens Runyon type II	*M. gordonae*	Pulmonary infections Skin infections Disseminated infections
Non-photochromogens Runyon type III	MAC	Pulmonary MAC infections Disseminated infections (mostly in AIDS patients) MAC associated lymphadenitis (in young kids and people with normal immune systems)
Rapid growing Runyon type IV	*M. abscessus* *M. chelonae* *M. fortuitum*	Skin and soft tissue infections (SSTI) Pulmonary infections Disseminated infections

Moreover, the study by Stahl and Urbance was pivotal in creating the first phylogeny for the Mycobacterium genus via *16S rRNA*, identifying a distinction between RGM and SGM, with RGM being more ancestral. However, limitations in *16S rRNA* gene resolution led to misclassifications, prompting the use of other markers, such as *hsp65* and *rpoB*, which offered better differentiation between closely related species. Genome sequencing has further refined Mycobacterium phylogeny, revealing distinct monophyletic groups and sparking debate over potential taxonomic revisions. Critics argue against dividing the genus on the basis of its clinical implications (Zhang et al., 2024).

Several studies in different countries have reported an increase in the prevalence of pulmonary diseases over time (Adjemian et al., 2012; Morimoto et al., 2014; Ide et al., 2015; Shah et al., 2016; Thomson et al., 2017). NTM were isolated from clinical specimens of hospitalized patients, which were sent to the Mycobacteriology Research Center (MRC) at Tabriz University of Medical Sciences (TUMS) for diagnosis.

One study investigated NTM infections in transplant patients in Iran. Researchers collected 57 respiratory samples from bone marrow and kidney transplant recipients and used culture methods and molecular techniques to identify NTM species. They reported an overall NTM prevalence of 22.8% in transplant patients. The most common species identified was the *Mycobacterium avium-intracellulare* complex (53.8% of isolates), followed by *M. marinum* (15.4%). Other species found included *M. xenopi, M. kansasii, M. simiae*, and *M. chelonae*. One isolate was identified as *M. tuberculosis*. The authors highlighted that NTM infections are an important risk factor for transplant patients, who are immunosuppressed and susceptible to opportunistic infections. They noted that timely detection and screening for NTM infections is critical in transplant wards, as many laboratories lack proper facilities or expertise to routinely identify these organisms. The high prevalence reported in this study indicates that NTM infections should be considered a serious concern for the health outcomes of transplant recipients. The authors recommend developing more routine methods to identify NTM infections in hospital settings that care for transplant patients (Mahdavi et al., 2021).

The prevalence of NTM infections has increased in patients with suspected tuberculosis. This study aimed to survey the genetic diversity of NTM species in patients with suspected tuberculosis in East Azerbaijan Province, Iran. Despite the importance of NTMs, no other research has been conducted in this region, making this the first study of their effects on NTMs in northwestern Iran.

## 2 Materials and methods

### 2.1 Collection of specimens and decontamination

This cross-sectional analysis focused on isolating non-tuberculous mycobacteria (NTM) from clinical specimens collected from patients suspected of having pulmonary tuberculosis. The specimens were collected over a period spanning from April 2021 to December 2022, totaling 20 months. A total of 230 clinical specimens (136 pulmonary and 94 extrapulmonary) were collected from suspected tuberculosis patients at health centers in East Azerbaijan Province, Iran. The pulmonary specimens consisted of 66.2% (90/136) sputum and 33.8% (46/136) bronchoalveolar lavage fluid (BALF). The extrapulmonary samples included 44.7% (42/94) of the skin samples, 36.2% (34/94) of the urine samples, and 19.1% (18/94) of the lymph node samples.

The suspected samples were decontaminated via the N-acetyl-L-cysteine 2% sodium hydroxide (NALC-2% NaOH) assay. Following decontamination, the sediments of the specimens were inoculated onto Löwenstein–Jensen medium and then incubated at 37°C for 8 weeks. Weekly monitoring was conducted to observe mycobacterial growth. The confirmation of suspected colonies as acid–alcohol–resistant bacilli was performed via the Ziehl-Neelsen staining technique (Bradner et al., 2013; Roshdi Maleki et al., 2019).

### 2.2 DNA extraction

To extract DNA, a loopful of mycobacterial cells was suspended in 500 μl of 1X Tris-EDTA buffer (10 mM Tris-HCl, 1 mM EDTA, pH 8.0) and heated at 80°C for 30 minutes to facilitate cell lysis. DNA was subsequently extracted via the cetyltrimethylammonium bromide (CTAB)/NaCl method, as described by van Soolingen et al (van Soolingen et al., 1991).

### 2.3 PCR for IS6110 and hsp65

The primers used (MTB1, 5′ CCTGCGAGCGTAGGCGTCGG 3′, and MTB2, 5′ CTCGTCCAGCGCCGCTTCGG 3′) amplified a 123-bp fragment of *IS6110*, which is specific for *M. tuberculosis* (Kabir et al., 2018). Samples that were not identified as *M. tuberculosis* by *IS6110* PCR were subjected to PCR for the detection of fragments of the *hsp65* gene (Telenti et al., 1993; Kim et al., 2005; Maleki et al., 2017). In this study, sequence analysis of the *hsp65* gene was used to identify NTMs (Kim et al., 2005). A 441-bp fragment of the *hsp65* gene was amplified with primer sets according to Telenti et al (Telenti et al., 1993).

PCRs were conducted using 10 mM Tris-HCl (pH 8.3), 50 mM KCl, 2 mM MgCl2, 0.2 mM dNTP mixture, 0.1 U/μl Taq polymerase, 0.5 μM each primer, the DNA template, and nuclease-free water. The PCR cycle conditions for amplifying the two genes (*IS6110* and *hsp65*) were as follows: 95°C for 4 min, followed by 30 cycles of 94°C for 30 s, 65°C for 30 s, and 72°C for 50 s, with a final extension at 72°C for 10 min. To ensure the accuracy of the PCR, DNA from *M. tuberculosis H37Rv* and nuclease-free water (Sinaclon, BioScience) were utilized as positive and negative controls, respectively.

Analysis of 3 μl of the PCR products (amplicon) was performed via electrophoresis on a 1.5% agarose gel. Following electrophoresis, the gel was stained with GelRed™ DNA stain, and the fragments were visualized under UV light using a gel documentation system (Gel Doc, ATP Co.).

### 2.4 Sequencing analysis

Sequence analysis of the *hsp65* gene was utilized for the molecular identification of clinical isolates. The PCR products were purified via a QIA quick PCR purification kit (QIAGEN, Germany) and then subjected to Sanger sequencing at Macrogen Corporation (Korea). The sequencing data for the *hsp65* gene were analyzed via DNASTAR Lasergene software (version 7.1).

The sequences were compared with similar sequences of the organisms in GenBank via BLAST online software from the National Center for Biotechnology Information (https://blast.ncbi.nlm.nih.gov/Blast.cgi). NTM species identification was confirmed if a 97% match was achieved (Mwangi et al., 2022).

To minimize the risk of false positives and negatives during PCR amplification, we performed biochemical tests on pure culture samples prior to molecular work. The PCRs were designed with specific primers verified through BLAST analysis to ensure accuracy. Positive and negative controls were included in each run, and all reactions were repeated twice to validate reproducibility and consistency (Gulvik et al., 2012).

### 2.5 Phylogenetic analyses

Molecular phylogenetic analyses were conducted via Molecular Evolutionary Genetics Analysis (MEGA) XI (version 11.0.13) (Tamura et al., 2021).

### 2.6 Submission of nucleotide sequence data to GenBank

Nucleotide sequences were submitted to GenBank to obtain accession numbers. The submission of nucleotide sequences was conducted through the web-based BankIt platform (https://www.ncbi.nlm.nih.gov/WebSub/), and GenBank staff assigned accession numbers upon receipt. The GenBank accession numbers for the nucleotide sequences can be found in **Table 3**.

**Table 3:** Distribution of NTM from clinical specimens with accession numbers.

NTM identified		Pulmonary isolates				Extrapulmonary isolates						Total (N & %)
		Sputum		BALF		Skin		Urine		Lymph node		
		N	ACNO	N	ACNO	N	ACNO	N	ACNO	N	ACNO	
**RGM**	*M. abscessus*	2	OR394794 OR394795	1	OR394796	0		0		1	OR394797	4 (19%)
	*M. chelonae*	0		0		1	OR394791	1	OR394792	1	OR394793	3 (14.3%)
	*M. fortuitum*	3	OR394783 OR394784 OR394785	0		1	OR394782	0		0		4 (19%)
	*M. mucogenicum*	1	OR394790	0		0		0		0		1 (4.8%)
**SGM**	*M. gordonae*	1	OR394780	0		1	OR394781	0		0		2 (9.5%)
	*M. kansasii*	2	OR389481 OR389482	0		0		0		0		2 (9.5%)
	*M. simiae*	3	OR389480 OR394786 OR394787	1	OR394788	0		1	OR394789	0		5 (23.8%)
**Total**		14 (66.7%)				7 (33.3%)						**21 (100%)**

BALF, bronchoalveolar lavage fluid; RGM, rapid-growing mycobacteria; SGM, slow-growing mycobacteria; ACNO, Accession number.

## 3 Results

**Figure 1:**
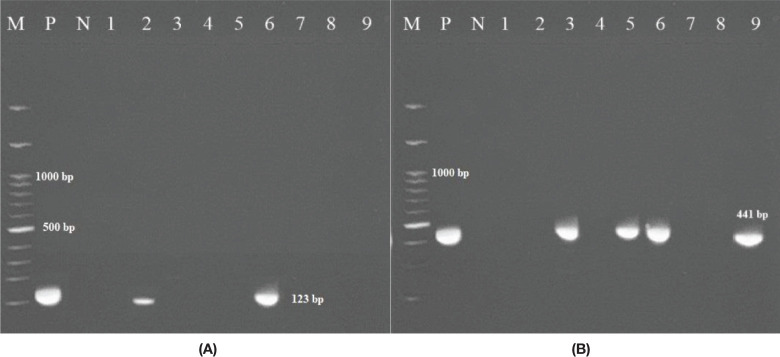
**(A)** Amplification of the 123 bp product of *M. tuberculosis* via PCR. The PCR products (amplicons) were analyzed via electrophoresis on 1.5% agarose gel. (M: represents a 100 bp DNA ladder; N: negative control; P: positive control DNA (*M. tuberculosis* strain *H37Rv*); 2, 6 clinically positive MTB samples; 1, 3, 4, 5, 7, 8, 9 non-MTB samples. **(B)** Figure to the right shows 441 bp of the *hsp65* gene (M: marker; P: positive; N: negative); 3, 5, and 6 clinically positive NTM samples.

Twenty-one NTM species were isolated from 230 clinical specimens (14 NTM from pulmonary specimens and 7 from extrapulmonary specimens) between April 2021 and December 2022. Of these, 12 (57.14%) were rapid-growing mycobacteria (RGM), and 9 (42.85%) were slow-growing mycobacteria (SGM). Overall, *M. simiae* (5/21, 24%) was the most frequently encountered species, followed by *M. abscessus* and *M. fortuitum*, each accounting for 4 out of the 21 isolates (19%). *M. chelonae* was present in 3 out of the 21 isolates (14.3%), while *M. kansasii* and *M. gordonae* were identified in 2 out of the 21 isolates (9.5%), and *M. mucogenicum* was found in 1 out of the 21 isolates (4.7%). Among the 230 clinical specimens, 2 (0.87%) were confirmed as part of the Mycobacterium tuberculosis complex (MTC) through biochemical and molecular testing. **Figure 1** displays the electrophoresis product of the 123 bp amplification of the *IS6110* sequence of MTC by PCR.

In the present study, we identified seven species of NTM in clinical specimens. *Mycobacterium avium* complex (MAC) was not detected in any of the specimens. **Table 3** presents the specifics of pulmonary and extrapulmonary clinically significant NTM isolates and their distribution among patients on the basis of specimen type. Notably, *M. mucogenicum* and *M. kansasii* were exclusively isolated from sputum samples.

As shown in **Table 3**, 66.7% (14/21) of the identified NTM species were of pulmonary origin, and 33.3% (7/21) were of extrapulmonary origin. Most species were isolated from sputum. Among the pulmonary samples, NTMs were identified in 12 (57.14%) sputum samples and two (9.5%) BALF samples. Among the extrapulmonary samples, NTMs were identified in three (14.3%) skin, two (9.5%) urine, and two (9.5%) lymph node samples.

**Figure 2:**
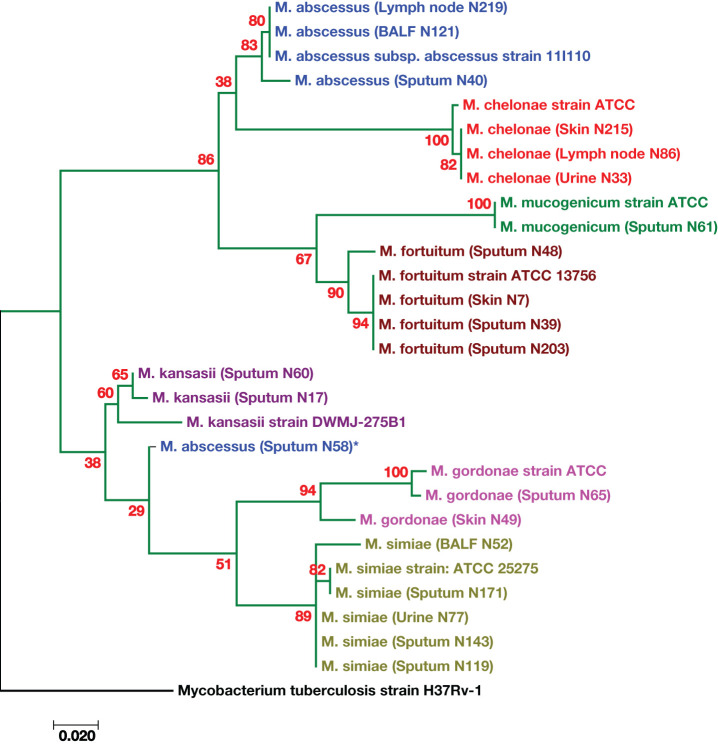
Phylogenetic tree based on the *hsp65* gene sequences of the isolates from patients with suspected tuberculosis in East Azerbaijan Province, northwestern Iran. A maximum likelihood tree was created, bootstrapped 100 times, and visualized with MEGA XI software (version 11.0.13) (Tamura et al., 2021). The *M. tuberculosis* strain *H37Rv* was used as the outgroup. The scale bar represents a 0.020 difference in nucleotide sequences.

Molecular phylogenetic and evolutionary analyses of the species, which were based on *hsp65* gene sequences, were performed via MEGA XI version 11.0.13 software (Tamura et al., 2021). A representative phylogenetic tree is shown in **Figure 2**. The *M. tuberculosis* strain *H37Rv* sequence was used as an outgroup. The *M. kansasii*, *M. gordonae*, and *M. abscessus* strains presented high genetic diversity (**Figure 2**). One strain of *M. abscessus* (obtained from sputum specimen No. 58) was genetically distinct from other identified *M. abscessus* species, as indicated by the star (*) in **Figure 2**.

## 4 Discussion

The increase in the number of outbreaks of NTM infections in patients with suspected TB is a major global challenge (Busatto et al., 2020). NTM infection is typically believed to be a result of exposure to environmental sources such as water, soil, and dust. According to studies conducted in Iran, nontuberculous mycobacteria account for 5% to 10% of mycobacterial infections (Nasiri et al., 2015). In this study, NTMs were identified in 9.13% (21/230) of patients with suspected tuberculosis, while only two MTB species were isolated from 230 clinical specimens. Therefore, the frequency of infections caused by nontuberculous species is higher than that caused by tuberculosis. The prevalence of NTM in the study conducted by Hoza et al. in Tanzania was also 9.7%, which is consistent with the results of the present study (Hoza et al., 2016). Furthermore, the prevalence of NTM in patients with suspected tuberculosis in the study conducted by Busatto et al. was 7% (Busatto et al., 2020). In this study, the diversity of NTM species was high, with 21 different species being isolated. Among these species, *M. simiae* was the most common, accounting for 23.8% of all the isolates. *M. simiae* is a prevalent NTM species in Iran and has recently been recognized as an emerging pathogen (Maoz et al., 2008; Hashemi-Shahraki et al., 2013; Heidarieh et al., 2016). Notably, *M. simiae* is the only niacin-positive NTM that can be mistaken for MTB because of similar clinical symptoms (Bhalla et al., 2018). These findings highlight the importance of investigating NTM species in patients suspected of having TB. Following *M. simiae*, *M. fortuitum* and *M. abscessus* were the next most frequently isolated species in our study. Similar findings have been reported in other studies, such as those conducted by Sharma et al (Sharma and Upadhyay, 2020). and Shenai et al (Shenai et al., 2010). in India, Albayrak et al. in Turkey (Albayrak et al., 2012), and Ahmed et al. in Pakistan (Ahmed et al., 2013). In Taiwan, Greece, and the United Kingdom, *M. fortuitum* is the second most commonly isolated NTM species after members of the *M. avium* complex (MAC), with percentages of 23%, 21%, and 20%, respectively (Hoefsloot et al., 2013).

The distribution of NTM species in this study, with *Mycobacterium simiae* (24%) being the most frequently isolated, aligns with reports from similar geographical regions. *M. simiae* has been documented as a prevalent cause of pulmonary infections, especially in immunocompromised individuals. The detection of *M. abscessus* and *M. fortuitum* in 19% of the isolates underscores the clinical importance of rapid-growing mycobacteria (RGM) in respiratory infections due to drug resistance challenges. Additionally, phylogenetic analysis of the *hsp65* gene revealed significant genetic diversity among these species, particularly among *M. abscessus* strains, suggesting potential intra-species variation (Tarashi et al., 2023).

In contrast to other studies that identified MAC as the dominant NTM species (Chalmers et al., 2020; Clarke et al., 2020), our study did not isolate any MAC species from the samples. Instead, we observed a prevalence of species such as *M. abscessus*, *M. chelonae*, *M. fortuitum*, *M. mucogenicum*, *M. gordonae*, *M. kansasii*, and *M. simiae* in East Azerbaijan, Iran.

A horizontal comparison of non-tuberculous mycobacteria (NTM) strains across Iran, Turkey, and China revealed both noteworthy similarities and differences in species prevalence, growth characteristics, species diversity, and transmission pathways. In Iran, a study identified 21 different NTM species from 230 clinical specimens, with *M. simiae* being the most prevalent at 24%. Other common species included *M. abscessus* and *M. fortuitum* (19% each), along with *M. chelonae* (14.3%). In Turkey, *M. gordonae* was the most isolated species, particularly in Samsun, whereas *M. abscessus* was more common in Istanbul and Malatya. Similarly, China reported *M. abscessus* as a leading species, especially among children with lower respiratory tract infections, which aligns with findings from both Iran and Turkey. The growth characteristics varied, with rapid-growing mycobacteria slightly more prevalent in Iran (57.14%) and particularly common in Turkey, where notable rapid growers included *M. abscessus* and *M. fortuitum*. Despite the global prominence of the *M. avium* complex, it was less frequently identified in these studies than rapid-growing species were.

The diversity of NTM species highlighted in Iran was significant, featuring a high prevalence of *M. simiae.* The Turkish study also noted diversity, particularly in Ankara, although it appeared more region-specific than Iran’s extensive variety. Chinese studies revealed regional variations, with *M. abscessus* and *M. chelonae* being frequently detected, but overall species diversity was somewhat lower than that in Iran. Environmental factors play crucial roles in NTM transmission across all three countries. In Iran, sources such as water, soil, and dust contribute significantly, with *M. simiae* being a common environmental contaminant complicating clinical diagnosis. Turkey echoed this trend, especially with *M. gordonae, which was* found due to its environmental ubiquity. Similarly, in China, *M. abscessus* and *M. fortuitum* are frequently linked to nosocomial infections. The clinical presentation of NTM infections often resembles that of tuberculosis, highlighting the necessity of accurate species identification in each region. This comparison underscores the complex, regionally diverse landscape of NTM species and emphasizes the need for tailored surveillance, diagnostic, and treatment strategies (Gunaydin et al., 2013; Liu et al., 2016).

Another study with similar findings was conducted by Gharbi et al (Gharbi et al., 2019). in Tunisia. They investigated the presence of NTM in specimens from individuals suspected of having pulmonary tuberculosis in northern Tunisia. In their study, they isolated nine species of NTM, namely, *M. kansasii*, *M. fortuitum*, *M. novocastrense*, *M. chelonae*, *M. gordonae*, *M. gadium*, *M. peregrinum*, *M. porcinum*, and *M. flavescens*. Like our study, they also did not isolate any MAC species from their samples. In the Ide et al. study (Ide et al., 2015) in Nagasaki, Japan, *M. intracellulare* was the most common pathogen. In the study by Mwangi et al (Mwangi et al., 2022). in Kenya, the dominant NTM species was the *Mycobacterium avium* complex. In the Mertaniasih et al. study (Mertaniasih et al., 2017), the most prevalent species were in the *M. avium-intracellulare* complex. In the Dadheech et al. study (Dadheech et al., 2023), *M. fortuitum* and *M. abscessus* were the most prevalent species. A study conducted across 30 predominantly European countries revealed that *M. xenopi* was most common in Hungary, the *M. abscessus* complex (MABC) was most common in South Korea and Taiwan, and *M. kansasii* was most prevalent in Poland and Slovakia (Hoefsloot et al., 2013).

A recent systematic review and meta-analysis examined the trends in the prevalence and antibiotic resistance of non-tuberculous mycobacteria (NTM) in China over the past two decades, emphasizing the growing importance of NTM infections in the context of tuberculosis (TB) management. An analysis of 339 publications revealed that *M. abscessus* and the *M. avium* complex (MAC) were the most prevalent NTM species, with a notable shift after 2015, when *M. intracellulare* became dominant. Despite an overall decrease in NTM prevalence, drug resistance patterns vary significantly among different species, complicating treatment, as NTM strains are generally sensitive to only a limited number of antibiotics, including ethambutol and linezolid. This study also highlighted regional differences in NTM species distributions linked to environmental and economic factors, underscoring the need for tailored treatment strategies given the variability in antibiotic resistance profiles (Zhou et al., 2020).

Another systematic review evaluating Whole Genome Sequencing (WGS) for detecting drug resistance in *M. tuberculosis* (MTB) synthesized data from 20 studies to compare WGS’s diagnostic accuracy with traditional phenotypic Drug Susceptibility Testing (DST), particularly for first-line drugs such as rifampicin and isoniazid. The review revealed that WGS has high sensitivity (0.98 for rifampicin and 0.97 for isoniazid), suggesting that it is a promising alternative to current DST methods. Despite its potential, the performance of WGS varies for other drugs, and challenges such as the need for standardized analytical pipelines, the complexity of interpreting mutation data, and inconsistent results for second-line drugs have been noted. The authors stressed the necessity for future research to incorporate clinical outcome data and improve the understanding of resistance mechanisms to increase the clinical relevance and reliability of WGS. While the findings highlight WGS’s promising capabilities for advancing TB diagnosis and treatment, particularly in high-income regions, further research and standardization are essential to fully harness its benefits (Papaventsis et al., 2017).

**Figure 3:**
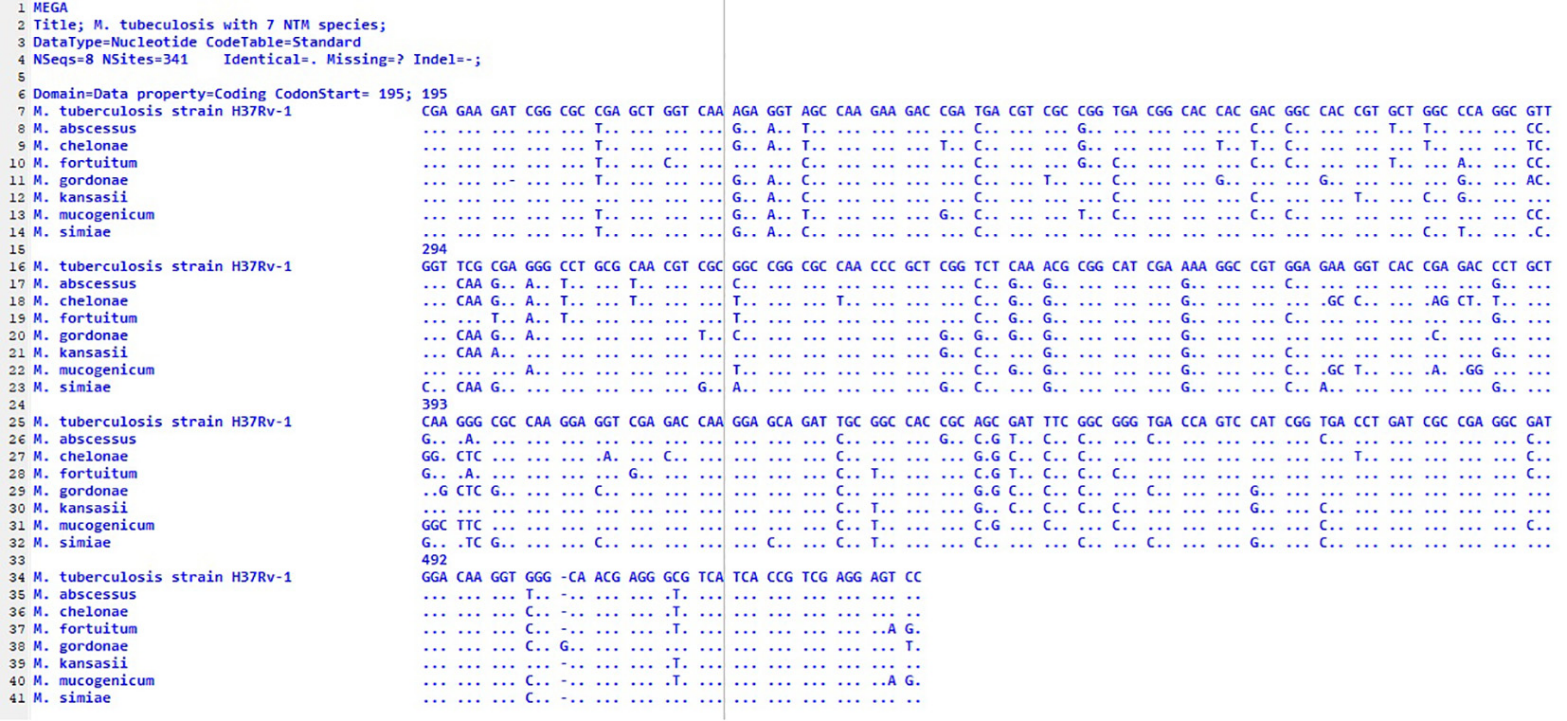
Alignment of partial sequences of the *hsp65* gene from seven different species. The *M. tuberculosis* strain *H37Rv1* was used because it differs from the MTB sequence; the dots indicate identity. The first nucleotide shown corresponds to position 195 of the published sequence from MTB. an out-group; nucleotides.

Therefore, it can be concluded that the distribution of NTM species varies across geographical locations (Hoefsloot et al., 2013). Our findings demonstrate regional variation in the diversity of NTM species and suggest the potential influence of different geographical or environmental landscapes within East Azerbaijan Province. NTMs frequently cause diseases that are clinically indistinguishable from tuberculosis (TB). Therefore, it is crucial to identify NTM species accurately, understand their distribution patterns, and determine the predominant species in different geographical regions. This is important because treatment strategies for NTM-related diseases vary. The *hsp65* gene, present in all mycobacteria, has more polymorphism than the *16S rRNA* gene sequence and is useful for identifying genetically related species (Kim et al., 2005). In this study, we used *hsp65* gene sequencing to identify nontuberculous mycobacteria. This molecular technique has previously been successful in differentiating NTM species (Kim et al., 2005; Escobar-Escamilla et al., 2014; Maleki et al., 2017). Our *hsp65* gene sequencing results confirmed that each species has a distinct and consistent sequence, as shown in **Figure 3**. The analysis presented in **Figure 3** fully characterizes the nucleotide differences along the length of the *hsp65* gene. Interestingly, these differences are particularly abundant in two regions (97–106 and 178–205), indicating that these regions are hypervariable regions of the *hsp65* gene.

**Figure 4:**
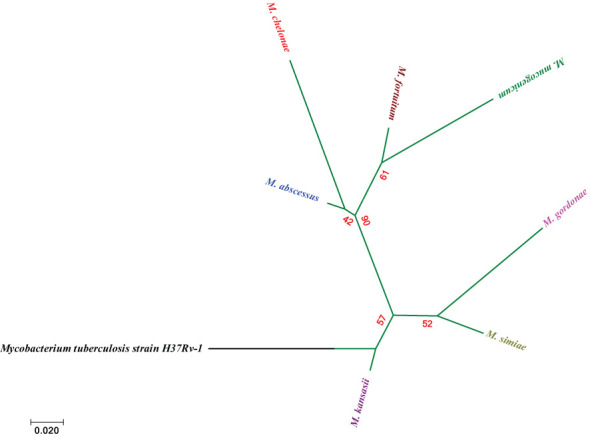
Molecular Phylogenetic analysis via the maximum likelihood method on the basis of *hsp65* sequences. The bar indicates an estimated 0.02 sequence divergence.

Among the rapidly growing mycobacteria, *M. mucogenicum* and *M. fortuitum* presented a high degree of similarity. On the other hand, *M. chelonae* and *M. mucogenicum* were the two RGM species with the greatest degree of difference. For the similarity and difference grades of the other species, please refer to **Figure 4**.

The genetic diversity revealed in this study, particularly within *M. abscessus*, highlights the potential for intraspecies variation that may have clinical relevance. While we employed *hsp65* sequencing to analyze this diversity, future studies could benefit from additional genotyping techniques, such as Multi-Locus Sequence Typing (MLST) to further elucidate genetic differences and improve our understanding of the epidemiology of NTM species (Alexander et al., 2014).

By employing both PCR and biochemical tests on pure culture samples, we minimized the risk of false results. The use of specific primers and multiple controls ensures the reliability of the PCR results (Kunduracılar, 2020).

Although Nested PCR was not used in the current study, it could be implemented in future studies to increase the sensitivity of detection, particularly in samples where low target concentrations may lead to false negatives. Given the high specificity of the primers used and the inclusion of quality control measures, we believe that our current approach was sufficient for the objectives of this study (Svec et al., 2015).

## 5 Conclusion

This study is the first to characterize the diversity of NTM in patients with suspected tuberculosis in East Azerbaijan Province. Twenty-one different NTM species were identified, with *M. simiae* being the most prevalent, followed by *M. fortuitum* and *M. abscessus*. Additionally, this study revealed a greater frequency of infections caused by nontuberculous species than by tuberculosis. These findings emphasize the importance of investigating NTM species in suspected TB patients. In TB-endemic areas, NTM-PD is often misdiagnosed as pulmonary tuberculosis, which can result in repeated treatment failures and an increased risk of complications. This highlights the need for better diagnosis.

## Data Availability

The datasets presented in this study can be found in online repositories. The names of the repository/repositories and accession number(s) can be found in the article/supplementary material.
